# Predictors of change in mental health and distress among women attending a women's shelter

**DOI:** 10.3402/ejpt.v5.24809

**Published:** 2014-09-12

**Authors:** Patricia Hoyeck, Kim Madden, Clare Freeman, Taryn Scott, Mohit Bhandari

**Affiliations:** 1 Division of Orthopaedic Surgery, Department of Surgery, McMaster University, Hamilton, Canada; 2 Department of Clinical Epidemiology & Biostatistics, McMaster University, Hamilton, Canada; 3 Interval House of Hamilton, Hamilton, Canada

**Keywords:** Intimate partner violence, mental health stages, DVSA stages of change, repeated shelter visits

## Abstract

**Background:**

Intimate partner violence (IPV) is detrimental to mental health. The Domestic Violence Survivor Assessment (DVSA), which includes a mental health assessment, is often used to evaluate abuse survivors in a counseling situation. The DVSA seeks to outline the cognitive state of women as per the stages of change as they attempt to move toward a life with no IPV.

**Objective:**

The objective of this study was to explore predictors of change in mental health and distress among women who entered a women's shelter more than once.

**Methods:**

Women entering a women's shelter more than once over a 3-year period were assessed by a trained social worker using the DVSA. A logistic regression analysis examined relationships between the chosen characteristics and the participants’ mental health through the DVSA stages of change.

**Results:**

We analyzed complete data for 94 women who entered the shelter a mean of 3.3 times (range 2–8) over a mean period of 16.1 days (range: 1–391). Thirty-six women (36/94; 38.3%) progressed through the stages. The average number of visits among women who progressed through the stages was 4. Our multivariable logistic regression showed women who had more visits to the shelter were almost twice as likely to progress through the stages compared to women who entered the shelter fewer times (OR=1.928; 95% CI=1.292–2.877; p=0.001). In the univariate analysis, only increased number of visits was significantly associated with progressing through the stages of change (OR=1.694; 95% CI=1.237–2.322; p=0.001). The other factors were not significantly associated with a change in mental health and distress (p>0.05).

**Conclusion:**

Women who enter women's shelters more frequently may be more likely to progress through the DVSA mental health stages compared to other women. Women's shelters may be helpful in assisting progression through the stages of change, thereby improving their mental health after abuse.

Intimate partner violence (IPV) is a prominent and significant societal issue. It is defined by the American Medical Association as “a pattern of coercive behaviors that may include repeated battering and injury, psychological abuse, sexual assault, progressive social isolation, deprivation, and intimidation” (AMA, cited in McCloskey, et al., 2007). Shelter systems are one form of support that may be available to women experiencing IPV and/or other forms of abuse. The use of shelter systems has increased since 2008 and the demand for services often exceeds shelter capacity (Burczycka & Cotter, 2011). Length of shelter stay is inversely related to re-abuse among women with posttraumatic stress disorder (PTSD), indicating that shelter visits can be successful in reducing abuse (Perez, Johnson, Johnson, & Walter, 2012).

Previous studies have indicated a clear connection between mental health and IPV. Women who experience IPV have a 3–5 times greater likelihood of depression, suicidality, PTSD, and addiction than non-victims (Adams, Sullivan, Bybee, & Greeson, 2008; Dutton et al., 2006). Abuse also has societal implications, as the majority of women who are currently labeled “offenders” by the legal system have been victim to physical and sexual abuse, many displaying symptoms of mental health disorders (Mood Disorders Society of Canada, 2009).

The data for this research came from a transition shelter, commonly defined as first-stage emergency housing. First-stage emergency housing is the first outlet of shelter a woman will move into upon escaping an abusive event. Second-stage emergency housing, a more permanent arrangement, may follow in some cases (Burczycka & Cotter, 2011). The programs the participating shelter offered were representative of other Ontario shelters. These services included safe residential emergency shelter services, outreach and legal advocacy services, a 24-hour crisis hotline, free emergency transportation, personal counseling, child and youth counseling programs, financial assistance, as well as referrals for medical attention, immigration information, and other community services.

The Domestic Violence Survivor Assessment (DVSA) has been used to assess abused women's cognitive state in order to tailor counseling services. The DVSA score, developed by Dienemann et al., seeks to outline the most relevant stages when a woman is attempting to move toward a life with no violence. The stages are 1) committed to continuing [the relationship], 2) committed but questioning [the relationship], 3) considering and preparing for change, 4) breaking away or partner reduces or ceases abusive behavior, and 5) establishes a new life—apart from their partner or together. Each of these stages is associated with a mental health and distress state described in Table 1.

**Table 1:** DVSA stages, descriptions, and typical answers for “How would you describe your mental health?”

Stage of change	Description	Typical responses on administered questionnaire
1 – Committed to continuing	Stressed, possibly depressed and confused.	“Overall it is good. I don't have any problems like that; I am just stressed a little.”
2 – Questioning commitment	Stressed/depressed, etc. May dislike self and have other symptoms.	“Some days are good, some days I feel stressed out, sad, nervous or have nightmares. I wish it would get better and I would be like myself again.”
3 – Considers change: abuse and options	High anxiety, panic attacks, fantasizes murder. Fears she is crazy.	“Not good, sometimes I fear I'm going crazy. I have some of these symptoms: always feeling scared, panic attacks, very sad, thinking of suicide or that killing him would be a way out, thinking and worrying about him all the time, jumpy and nervous, can't sleep, nightmares or sleeping too much, not hungry or hungry all the time.”
4 – Breaks away or partner curtails	Senses she can gain control of “out of control” feelings.	“It's not good, but I know I am not crazy, just under a lot of stress from the abuse. Some days I can control my reactions and others are not so good yet.”
5 – Establishes a new life apart or together	Grief rises then recedes. Lower self esteem slowly improves.	“Six or more months with no abuse and it is slowly getting better.”

From Dienemann et al., 2002.

## Objective

The objective of this study was to explore predictors of change in mental health and distress among women who entered the women's shelter more than once, as per the DVSA mental health stages of change. Potential predictors that we chose to explore included abuser use of alcohol, monthly income, immigration status, relationship to the abuser, number of visits to the shelter, and type of abuse. Though not inclusive of all potential predictors, these findings may assist shelters in identifying women who may be in need of greater mental health support. Improved identification could facilitate targeted interventions in order to better assist these women.

## Materials and methods

### Study design

We conducted a retrospective database study of 286 women who entered a first-stage emergency shelter more than once over a period of 3 years. We evaluated their change in mental health and distress using the DVSA scale. The data for this research were collected upon entrance to the shelter during a 3-year study period and again at any subsequent visit during the study period. Women completed four questionnaires administered by an experienced social worker. These questionnaires ranged from gathering demographic information to assessing stages of change.

### Ethics and confidentiality

We received approval from the Hamilton Integrated Research Ethics Board (Project 12–153) before beginning this study. At the time of entering the shelter, women provided informed consent to enter their de-identified information into a database for research use. Research personnel had access to coded and de-identified data only. Confidentiality was maintained at all times and only aggregate data were used for this analysis.

### Participants

This study aimed to have minimal exclusion criteria. Women older than 16 who entered the shelter more than once over the 3-year study period could be included in the study. Those women who chose to participate completed the DVSA assessment and consented to their data being used for research purposes.

### Intake assessment

A general demographics information form and an interview intake form were collected for each woman who entered the local women's shelter. A total of 286 women were interviewed and assessed by an experienced social worker using the DVSA, which included the mental health assessment. The DVSA consisted of 13 categories as shown in Table 2 (Dienemann, Glass, Hanson, & Lunsford, 2007).

**Table 2:** DVSA categories

DVSA category
Triggers of abusive incidents
Managing partner abuse
Seeking legal sanctions
Attachment
Views relationship and considers options
Managing loyalty to norms and own beliefs
Accessing help
Self-identity
Self-efficacy
Personal feelings
Mental health
Control of money
Medical care

From Dienemann et al., 2007.

### Measurement of mental health

All women entering the women's shelter were interviewed by an experienced social worker who used the DVSA to assess women's cognitive states, specifically mental health and distress as per the mental health stages of change. The women discussed their situation with the trained social worker who recorded the most appropriate stage of change based on the woman's description of her mental health and distress. If women identified as transitioning between two stages, an average of the scores were taken. The typical answer at each stage, as scripted on the administered questionnaire, is outlined in Table 1. The DVSA did not measure mental health and distress linearly. In other words, Stage 2 represented less mental distress than Stage 3, but Stage 3 was further along the progression of stages than Stage 2, paradoxically. This is because deciding to leave a partner and actually leaving a partner are stressful and sometimes dangerous. The mental distress begins to resolve at Stage 4 and is markedly improved at Stage 5.

We assessed characteristics that may theoretically influence changes in mental health and distress as measured by the DVSA stages. We included six potential predictor factors that were available to us in the database: abuser use of alcohol, monthly income (less than $1,000 or more than $1,000), immigration status (Canadian citizen/permanent resident or other), relationship to the abuser (partner or other), number of visits to the shelter, and whether the woman experienced physical or sexual abuse. Nearly all women experienced non-physical forms of abuse so we did not include this as a predictor variable.

### Outcome

The primary outcome was change in mental health and distress as measured by the DVSA stages. Women were interviewed by a trained social worker who after an extensive interview, identified women to be in one of the five stages of change. We determined whether women progressed through the stages by comparing their stage of change at the first visit to their stage of change at the last visit, coding this as a binary variable (progressed vs. did not progress).

### Data analysis

Descriptive data are presented as frequency counts and percentages or means and standard deviations for continuous data. The primary outcome was analyzed using a multivariate logistic regression model, presented as odds ratios with 95% confidence intervals. We also conducted univariate logistic regressions for each predictor variable. All data were analyzed with SPSS version 22.0.

## Results

**Fig. 1:**
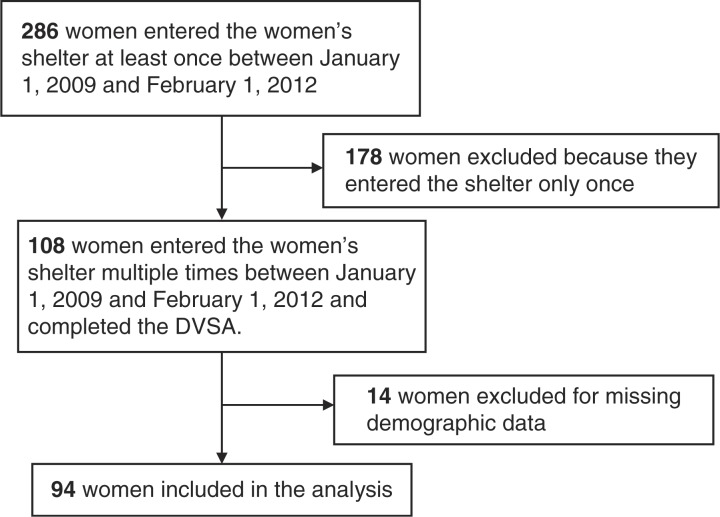
Study flow diagram.

We collected data for 286 eligible women (535 visits). 108 of these women entered the women's shelter more than once over the 3-year study period; 14 women were excluded due to missing demographic data. We included 94 women in our analysis as shown in Fig. 1.

### Participant characteristics

Table 3 provides a summary of the characteristics of women included in the analysis. Abusers often used alcohol (34.3%). Many women had a monthly income of under $1,000 (85.3%), were Canadian citizens or permanent residents (50.9%), and were abused by their partner (70.4%) as opposed to family or friends. The most common types of abuse the women experienced were verbal (88.0%), emotional (88.0%), psychological (85.2%), and physical abuse (65.7%).

**Table 3:** Participant characteristics

Characteristic		n	%
Abuser uses alcohol	Yes	37	34.3
	No	71	65.7
	<$1,000	93	85.3
Monthly income	≥$1,000	15	13.8
	Missing	1	0.9
Immigration status	Canadian citizen/permanent resident	55	50.9
	Other	54	49.5
	Partner	76	70.4
Abuser's relationship	Other	18	16.7
to victim	Missing	14	13.0
	Mean 3.31		
Number of visits	Standard deviation 1.46		
	Range 2–8		
Type of abuse		n	%
Verbal abuse	Yes	95	88.0
	No	13	12.0
Emotional abuse	Yes	95	88.0
	No	13	12.0
Psychological abuse	Yes	92	85.2
	No	3	2.8
	Missing	13	12.0
Physical abuse	Yes	71	65.7
	No	24	22.2
	Missing	13	12.0
Financial abuse	Yes	64	59.3
	No	31	28.7
	Missing	13	12.0
Sexual abuse	Yes	30	27.8
	No	65	60.2
	Missing	13	12.0
Spiritual abuse	Yes	10	9.3
	No	59	54.6
	Missing	39	36.1

The participants used the shelter a mean of 3.3 times over a period of 16.1 days (range 1–391 days). The mean change in mental health and distress was a progression of 0.32 stages over this time period (range −1.5 to +2). Thirty-six women (36/94; 38.3%) progressed through the mental health stages of change over the duration of the study. The average number of visits among women who progressed through the stages was 4 in comparison to 2.8 for women who showed no progression. Two participants experienced a negative progression in their DVSA mental health score; one was initially in the fourth stage of change, and regressed to the third stage, while the other participant started between the second and third stage, and regressed to the first. These women attended the shelter system three times and four times, respectively.

### Primary analysis

**Fig. 2:**
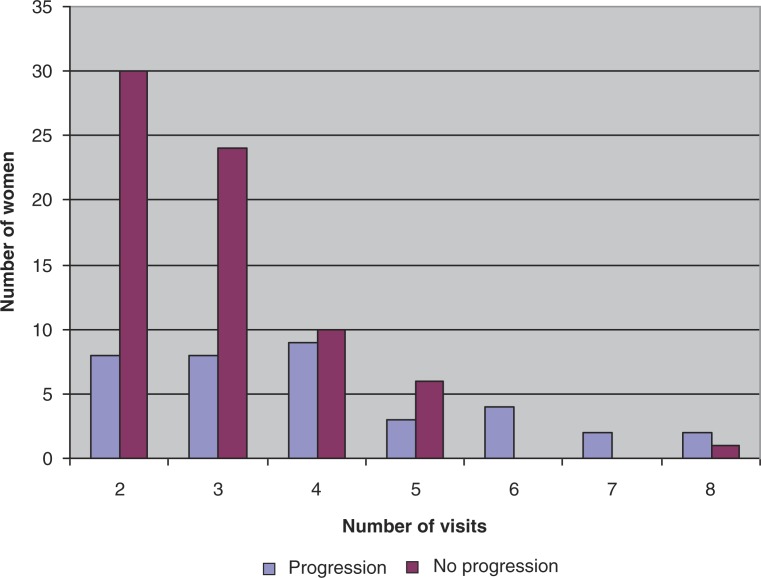
Progression through the stages by number of visits.

Our multivariable logistic regression showed women who had more visits to the shelter were almost twice as likely to progress through the mental health stages compared to women who entered the shelter fewer times (OR=1.928; 95% CI=1.292–2.877; p=0.001). Women who were abused by a partner as opposed to a family member or friend were more than 5 times as likely to progress through the mental health stages but this did not reach statistical significance (OR=5.167; 95% CI=0.0867–30.797; p=0.071). In the univariate analysis, only increased number of visits was significantly associated with progressing through the stages of change (OR=1.694; 95% CI=1.237–2.322; p=0.001). The other factors (monthly income, immigration status, abuser use of alcohol, and type of abuse) were not significantly associated with change in mental health and distress (p>0.05) (Table 4). Figure 2 graphically illustrates these results; women were less likely to progress through the mental health stages of change when they entered the shelter fewer times.

**Table 4:** Logistic regression analysis for progression through the mental health stages

Characteristic	Univariate		Multivariate	
	
	Odds ratio (95% CI)	p	Odds ratio (95% CI)	p
Increased number of visits	1.694 (1.237–2.322)	0.001	1.928 (1.292–2.877)	0.001
Abused by spouse/partner	1.167 (0.481–2.831)	0.732	5.167 (0.0867–30.797)	0.071
Monthly income >$1,000	0.682 (0.201–2.314)	0.539	1.389 (0.281–6.869)	0.687
Abuser uses alcohol	1.905 (0.829–4.376)	0.129	2.265 (0.802–6.395)	0.123
Physical/sexual abuse	2.891 (0.773–10.807)	0.115	1.770 (0.381–8.219)	0.466
Non-citizen	1.439 (0.643–3.223)	0.376	1.477 (0.453–4.819)	0.518

## Discussion

These results attempted to establish what factors can be reliably used to determine a woman's DVSA mental health stage according to several chosen characteristics. The results indicated that women from a variety of backgrounds are impacted by IPV, demonstrating that it is not restricted to certain groups. Furthermore, in a sample of 94 participants, we found that women who entered the women's shelter more frequently during the study period were almost twice as likely to progress through the mental health stages compared to other women. As can be seen by the responses in Table 1, though the hope is for women to reach a higher stage of change, improvement for the question, “How would you describe your mental health?” is not linear. Therefore, prior to mental health improvements, change-associated difficulties are faced.

A possible explanation for the finding that women who entered the women's shelter more frequently were twice as likely to progress through the mental health stages is that these women were taking steps to resolve their violent situation. It is possible that the shelter helped women progress through the stages by offering support, mental health counseling, resources, and showing the women that they were not alone. Two participants had a negative progression over their visits as they were initially in a more advanced stage and found themselves in an earlier stage by their last shelter visit. Thirty-six women did show increasing scores. Women who progressed had an average of 4 visits to the shelter compared to 2.8 visits for women who did not show progression. This further supports the findings of the regression analysis—progression often results upon multiple visits to the shelter.

Monthly income, type of abuse, relationship to the abuser, immigration status, and abuser use of alcohol showed no significant association with improvement in the mental health stages. Though these factors are important in assessing the stability of one's lifestyle, they did not lead to improvement in a woman's mental health as per the DVSA for the women in this study. Predictors such as length of stay, and time between visits may have also been significant, however were not regressed due to missing data. These factors may have provided a further understanding of the dynamics between shelter visits and mental health improvements.

Previous studies have indicated that financial need and alcohol are common stressors among women experiencing violence (Adams et al., 2008; Foran & O'Leary, 2008). It was found that the most common stressor revolved around financial matters (Sutherland, Bybee, & Sullivan, 2002). Furthermore, a meta-analysis revealed that alcohol use is associated with perpetrating IPV (Foran & O'Leary, 2008). Previous studies have also shown that immigration status is not necessarily associated with IPV, but mental health can be negatively affected by IPV and is significantly worse when women have immigrated (Du Mont et al., 2012; Wong, Tiwari, Fong, Humphreys, & Bullock, 2011). Potential reasons for the discrepancy between our results and the literature are that the DVSA score utilized for this study was very specific in terms of IPV and mental health; all responses related directly to feelings within the relationship. Furthermore, bias could have resulted from the small sample size.

## Strengths and limitations

A strength of this study is that a trained and experienced social worker rigorously assessed the outcome variable (DVSA mental health stage). Furthermore, placing minimal exclusion criteria allowed for a diverse group of study participants who were representative of the population of women's shelter users in a medium-sized city in Canada. However, these results could be strengthened by a larger sample size and by including more than one site in the data collection. This being a retrospective database study, we were limited by a set number of participants and could therefore not accurately test the differences in progression at later visits. A true comparison between the fourth, fifth, and sixth visits was not possible. As with many database studies we were limited by the variables that were already collected and could not assess other factors that were not collected as part of the routine intake assessment. For future studies, it would be interesting to use a prospective design to assess mental health directly as opposed to through stages of change.

## Conclusion

Women who enter women's shelters more frequently may be more likely to progress through the mental health stages compared to women who attend less frequently. Women's shelters may be helpful in assisting women in their progression through the stages of change, thereby improving their mental health after abusive events.
